# *Psallusthomashenryi* sp. n. and *Psalluslucanicus* from Turkey (Hemiptera, Heteroptera, Miridae)

**DOI:** 10.3897/zookeys.796.21536

**Published:** 2018-11-15

**Authors:** Attilio Carapezza, Petr Kment

**Affiliations:** 1 University of Palermo; corresponding address: via Sandro Botticelli 15, 90144 Palermo, Italy University of Palermo Palermo Italy; 2 Department of Entomology, National Museum, Cirkusová 1740, CZ-193 00 Prague 9 – Horní Počernice, Czech Republic Department of Entomology, National Museum Prague Czech Republic

**Keywords:** Hemiptera, Heteroptera, Miridae, new record, new species, Palearctic region, Phylinae, Phylini, *
Psallus
*, Turkey

## Abstract

Psallus (Psallus) thomashenryi**sp. n.** (Hemiptera: Heteroptera: Miridae: Phylinae: Phylini: Phylina) is described from southern Anatolia, Turkey. Illustrations of the dorsal habitus and male genitalia are provided. Its habitus is similar to other uniformly orange species of the subgenus Psallus Fieber, 1858, particularly *P.asthenicus* Seidenstücker, 1966 from which it can be easily distinguished by the combination of extremely small size (2.3 mm in both sexes) and different morphology of the vesica. Psallus (Psallus) lucanicus Wagner, 1968 is recorded for the first time from Turkey. Psallus (Psallus) aurora (Mulsant & Rey, 1852) is removed from the list of Turkish fauna based on a reevaluation of the voucher specimen. An updated checklist of the species of *Psallus* known to occur in Turkey is provided. The relevance of Anatolia and the Syro-anatolian-transcaucasian region in the Palearctic distribution of *Psallus* is discussed. The westernmost record of another mirid, *Plagiognathusmarivanensis* Linnavuori, 2010, is provided.

## Introduction

The predominantly Palearctic genus *Psallus* Fieber, 1858, including 160 valid species-group taxa, is one of the most speciose in the subfamily Phylinae (Schuh 1995, 2013; Kerzhner and Josifov 1999; Yasunaga 2010; Matocq 2011; Duwal et al. 2012; Vinokurov and Luo 2012; Aukema et al. 2013; Li and Liu 2013; Schuh et al. 2014; Simon and Strauss 2014; Konstantinov 2016; Pagola-Carte 2017). *Psallus* is currently subdivided into eight subgenera based on similarities in habitus and male genitalia: *Apocremnus* Fieber, 1858, *Calopsallus* Yasunaga & Vinokurov, 2000, *Hylopsallus* Wagner, 1952, *Mesopsallus* Wagner, 1970, *Psallus* Fieber, 1858, *Phylidea* Reuter, 1899, *Pityopsallus* Wagner, 1952, and *Supsallus* Linnavuori, 1993 (Kerzhner and Josifov 1999, Yasunaga and Vinokurov 2000, Yasunaga 2010). Some authors have regarded this division into subgenera and the definition of the genus itself far from satisfactory and in need of revision; the genus is generally considered polyphyletic and several proposals have been made to combine some subgenera, elevate some subgenera to genus level, and/or transfer some species to other genera (Yasunaga and Vinokurov 2000, Wyniger 2004, Mróz 2012, Aukema et al. 2013, Schuh and Menard 2013, Pluot-Sigwalt and Matocq 2017). Probably a better understanding of the systematics of *Psallus* will be reached only by dealing with it on a world basis and extending the use of female genitalia, whose relevant value in aiding the recognition of related and unrelated species was recently demonstrated by Pluot-Sigwalt and Matocq (2017).

Prior to this study, 34 species of *Psallus* were known to occur in Turkey. Our study documents two additional species; the first of them, Psallus (Psallus) thomashenryi sp. n., is described from Southern Anatolia, and the second, Psallus (Psallus) lucanicus Wagner, 1968, is recorded for the first time from Turkey. The West-Mediterranean Psallus (Psallus) aurora (Mulsant & Rey, 1852) is removed from the list of Turkish fauna based on a reevaluation of the voucher specimen.

## Material and methods

Images of the adults were taken using a Canon D40 camera equipped with a MP-E65 macro lens mounted on a photographic stand; stacked images were combined using Zerene Stacker. Drawings of 10 % KOH-macerated genitalia were made using a Leitz Laborlux S microscope equipped with camera lucida. Measurements were made using an eyepiece micrometer mounted on a Wild M5S binocular microscope. All measurements are in millimeters. Morphological terminology follows Schuh and Slater (1995); terminology of male genitalia follows Konstantinov (2003).

In the transcription of locality labels of types a slash (/) is used to indicate data in different rows of a single label; a double slash (//) is used to separate different labels; data on the labels are given verbatim.

All specimens mentioned in the text are deposited in the National Museum, Prague, Czech Republic (**NMPC**).

## Taxonomy

### Psallus thomashenryi sp. n.

Taxon classificationAnimaliaHemipteraMiridae

http://zoobank.org/EDE2EE97-C112-4896-9AC1-A805CF5A9AB4

Figs 1 , 2–7

#### Type locality.

Turkey, southern Anatolia, Mersin Province, Göksu Nehri river canyon, Evkafçiftliği, 36°27'23.6"N, 33°38'12.3"E.

#### Type material.

**Holotype**: ♂, glued on a pointed cardboard with genitalia glued on the same cardboard with labels as follows: 36°27'23.6"N, 33°38'12.3"E / AS. TURKEY, İÇEL prov. / Evkafçiftliği, Göksu Nehri canyon / valley of drying brook, sweep / 5.v.2007, lgt. P. Kment [white printed label] // HOLOTYPUS / *PSALLUS (PSALLUS)* / *THOMASHENRYI* / sp. n. / det. Carapezza & Kment 2017 [red printed label]’ (NMPC).

**Paratype**: ♀, glued on a pointed cardboard with labels as follows: 36°27'23.6"N, 33°38'12.3"E / AS. TURKEY, İÇEL prov. / Evkafçiftliği, Göksu Nehri canyon / valley of drying brook, sweep / 5.v.2007, lgt. P. Kment [white printed label] // PARATYPUS / *PSALLUS (PSALLUS)* / *THOMASHENRYI* / sp. n. / det. Carapezza & Kment 2017 [red printed label]’ (NMPC).

#### Description.

**Figure 1.**
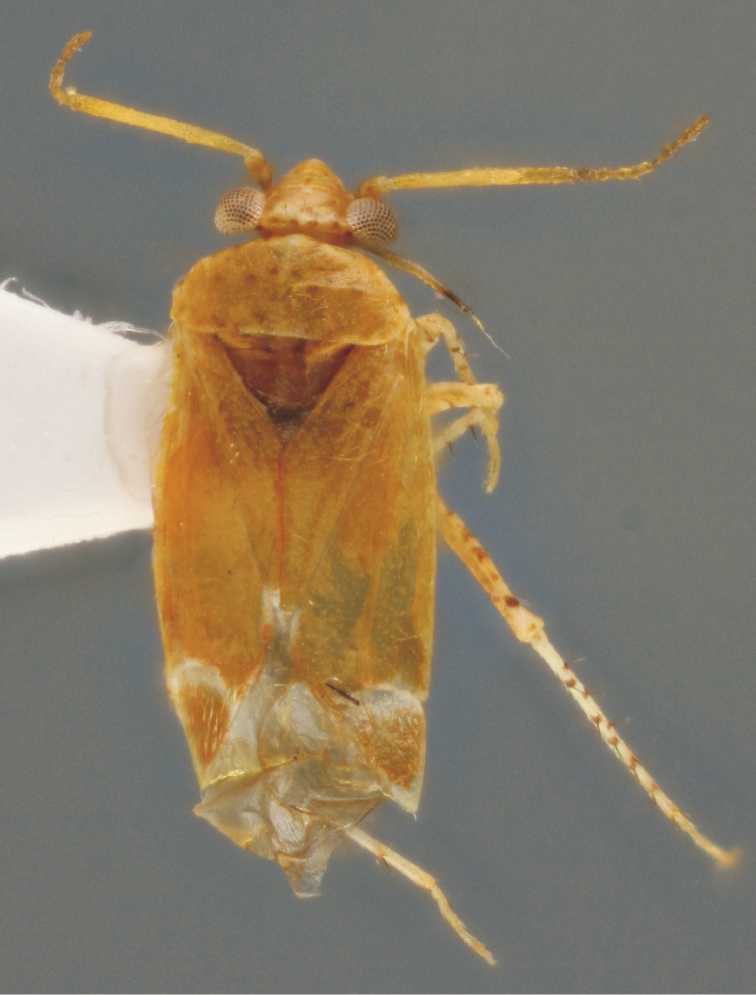
Habitus of *Psallusthomashenryi* sp. n., holotype, male (2.29 mm).

***Male***. *Coloration* (Fig. 1). Dorsal coloration almost uniformly orange. Head orange, vertex basally with four small reddish dots arranged in line, frons with five whitish lateral arcs; apex of clypeus whitish. Antennae pale yellowish, scape with faint basal annulation and with two preapical dark dots; labium pale yellowish, apical half of last segment darkened. Pronotum orange with traces of reddish dotting in anterior half; scutellum and hemelytra orange, cuneus basally and apically whitish; membrane pale, hyaline, veins concolorous. Thoracic sterna orange with reddish tinge, legs pale yellowish, femora with irregular orange to reddish-brown dots, more numerous on hind femora; tibial spines black, arising from small dark spots; tarsi uniformly pale.

**Figure 2–7.**
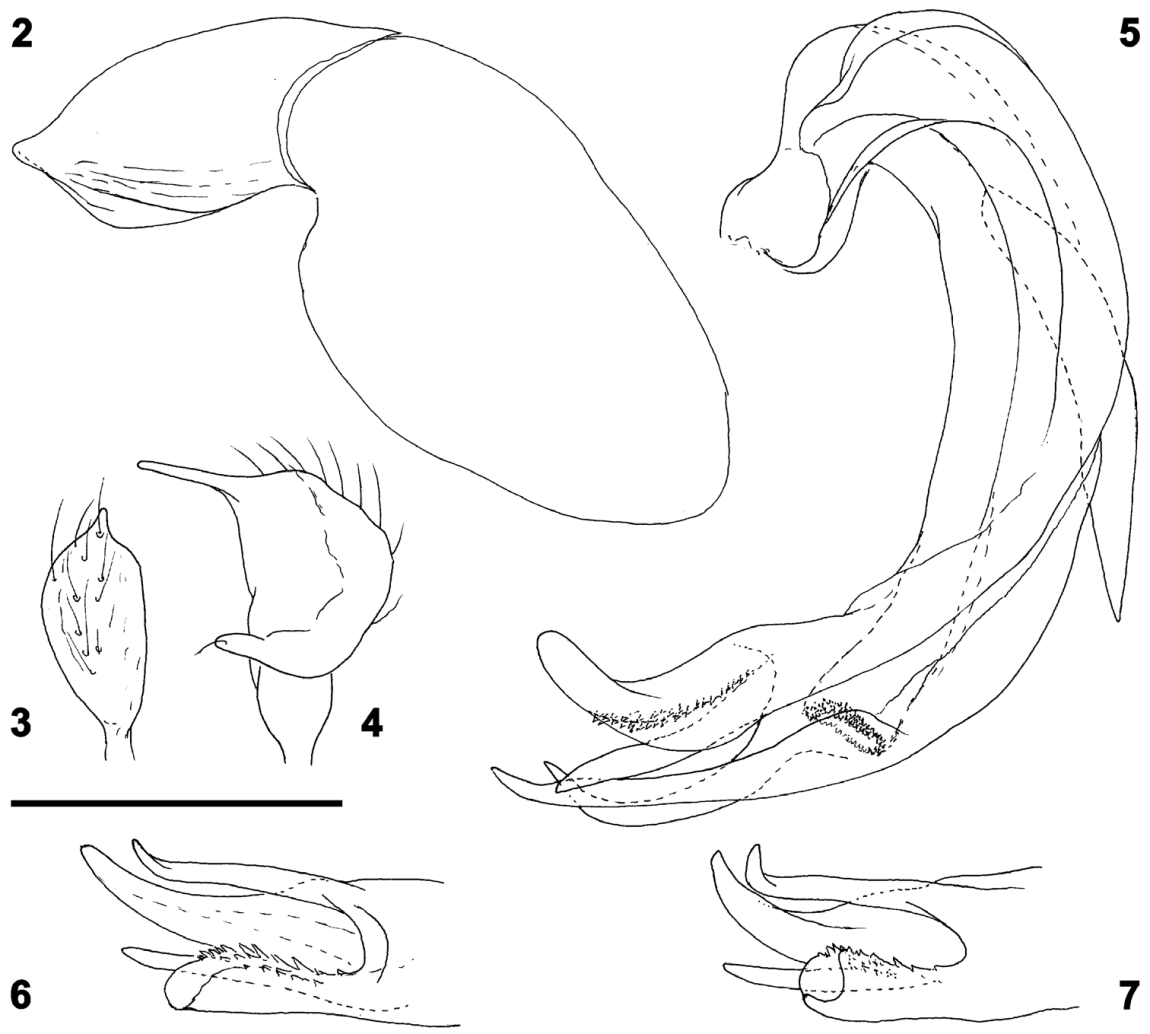
Male genitalia of *Psallusthomashenryi* sp. n.: **2** phallotheca **3** left paramere **4** right paramere **5** vesica in lateral view **6–7** apex of vesica in different views. Scale bar: 0.2 mm.

*Structure.* Body elongate-ovoid (Fig. 1), about 2.8 times longer than basal width of pronotum. Head moderately projecting, in dorsal view 2.1 times wider than long, in frontal view 1.5 times wider than high, in lateral view 1.5 times longer than high; ocular index (ratio vertex/eye in dorsal view) 1.6. Antennae with segment II 0.8 times as long as basal width of pronotum. Labium slightly surpassing metacoxae. Hind femora elongate, 3.6 times longer than maximum width; tibial spines long, about twice longer than tibial diameter. Genital segment ventrally unkeeled; phallotheca (Fig. 2) robust, with a preapical lateral ridge, apex rounded; left paramere (Fig. 4) broad, apical process straight and thin, sensory lobe short, apically rounded; right paramere (Fig. 3) elongate, apical process straight; vesica (Figs 5–7) short, C-shaped, provided with robust postbasal lateral spicule extending apically to middle of vesica, terminating in elongate, apically recurved blade, armed with rows of denticles along inner side, and three fingerlike, apically bent blades, almost equal in size, originating near subapical secondary gonopore.

*Pubescence.* Dorsum with reclining pale and semierect blackish setae; the latter few, mostly on head and lateral margins of pronotum.

***Female.*** Coloration similar to males but paler. Structure and pubescence as in males, but body more ovoid, 2.8 times longer than basal width of pronotum; ocular index 2.2. Female genitalia could not be examined due to the imperfectly sclerotized single specimen.

#### Measurements

(in mm). *Male.* Body length: 2.29; head width: 0.61; interocular distance: 0.27; pronotum width: 0.82; length of antennal segments: I – 0.13, II – 0.63, III – 0.29, IV – 0.24; length of tarsomeres: I – 0.11, II – 0.13, III – 0.15. *Female*. Body length: 2.38; head width: 0.61; interocular distance: 0.32; pronotum width: 0.89; length of antennal segments: I – 0.14, II – 0.58, III and IV missing.

#### Differential diagnosis.

The dorsal coloration almost uniformly orange and the C-shaped vesica with elongate apical processes show clearly that the new species belongs to the subgenus Psallus s. str. Its total length, 2.3 mm in both sexes, makes it one of the smallest species in the subgenus; only a few species have a body length less than or equal to 2.5 mm, namely *P.corsicus* Puton, 1875 and *P.jeitensis* Wagner, 1963, but their coloration and male genitalia differ from those of the new species. By its habitus, *Psallusthomashenryi* is very close to the East-Mediterranean *P.asthenicus* Seidenstücker, 1966, from which, as from any other species of its genus, it can be distinguished by the characteristic male genitalia, especially the unique apical blades of the vesica. In particular, *P.asthenicus* is larger (body length 2.8–3.1 mm), the postbasal lateral spicule of the vesica is membranous and its apical blades are horn-like, gradually tapering, apically pointed, and the central one is shaped like the head of a bird (see Seidenstücker 1966, figs 25a, 25b).

#### Etymology.

The new species is named in honor of our colleague Thomas J. Henry on his 70th birthday in recognition of his great contribution to the advancement of heteropterology and as a token of personal friendship and gratitude. The specific epithet is a noun in the genitive case.

#### Habitat.

The specimens were beaten from shrubs and trees growing around a small drying-up brook at the village margin. In the same habitat, the new species was collected with the following other species of Miridae: *Amblytylusconcolor* Jakovlev, 1877, *Closterotomusannulus* (Brullé, 1832), *C.norwegicus* (Gmelin, 1790), Globiceps (Paraglobiceps) syriacus Wagner, 1969, Heterocordylus (Bothrocranum) carbonellus Seidenstücker, 1956, *Lepidargyrussyriacus* (Wagner, 1956), *Paredrocorispectoralis* Reuter, 1878, Phytocoris (Exophytocoris) parvulus Reuter, 1880, and *Plagiognathusmarivanensis* Linnavuori, 2010.

#### Distribution.

Endemic to southern Anatolia.

### Faunistic records and corrections

#### Atomoscelis onusta

Taxon classificationAnimaliaHemipteraMiridae

(Fieber, 1861)

= Psallusaurora (misidentification): Hoberlandt (1956): 54 (record).

##### Material examined.

**TURKEY: Anatolia: Adana Province**: Toros Mts., Kozan, 8.–9.viii.1947, 1 ♂, Exp. N. Mus. ČSR lgt., L. Hoberlandt 1954 det. as *Psallusaurora* (NMPC).

##### Comment.

*Psallusaurora* is a West-Mediterranean species known from France, Italy, Portugal, Spain, Algeria, Libya, Morocco and Tunisia (Kerzhner and Josifov 1999). The record from Turkey was considered doubtful by Kerzhner and Josifov (1999: 412). Our recent reexamination of the voucher specimen confirmed that it was misidentified and belongs to the widely distributed Palearctic species *Atomoscelisonusta*. We therefore exclude *P.aurora* from the list of Turkish fauna.

#### Plagiognathus (Plagiognathus) marivanensis

Taxon classificationAnimaliaHemipteraMiridae

Linnavuori, 2010

Plagiognathus (Plagiognathus) marivanensis Linnavuori, 2010: 388 (original description).

##### Material examined.

**TURKEY: Anatolia: Mersin Province**: Göksu Nehri river canyon, Evkafçiftliği, 36°27'23.6"N, 33°38'12.3"E, valley of drying brook, sweeping, 5.v.2007, 1 ♀., P. Kment lgt. (NMPC).

##### Distribution.

The species was described recently from western Iran (provinces Kohgiluyeh and Boyerahmad, Kurdestan, and West Azerbaijan) by Linnavuori (2010). It was later recorded from Turkey, eastern Anatolia (provinces Elazığ and Dıarbakır; Matocq et al. 2014). Here the westernmost record of this poorly known species is presented, extending its distribution to southern Anatolia.

#### Psallus (Psallus) lucanicus

Taxon classificationAnimaliaHemipteraMiridae

Wagner, 1968

Psallus lucanicus Wagner, 1968: 273 (original description).
Psallus balcanicus Josifov, 1969: 29 (original description). Synonymized by Carapezza (1988: 118, suspected) and Kerzhner and Josifov (1999: 416, confirmed).

##### Material examined.

**TURKEY: Anatolia: Mersin Province**: Yeniköy env., slope above road to Gözne, 36°59'18.5"N 34°30'19"E, on Quercuscf.cerris, 6.v.2007, 2 ♂♂ 3 ♀♀, P. Kment lgt. (NMPC).

##### Host plant.

It is generally collected on *Quercuscerris* (Carapezza 1988, Bryja and Kment 2002, Rabitsch 2003, Anonymus 2016, Denton 2016), but it also is known from *Q.pubescens* (Carapezza 1988) and *Q.macrolepis* (Rieger 2007).

##### Distribution.

The species was described from Lucania, a region in Southern Italy whence its name is derived. It was later found in other Italian regions including Sicily (Wagner 1968, Carapezza 1988, Wyniger 2004), and in Austria (Rabitsch 2003), Bulgaria (Josifov 1969, as *P.balcanicus*; Wyniger 2004), Czech Republic (Bryja and Kment 2002, Wyniger 2004), Greece (Rieger 2007), Hungary (Kondorosy 2005), Slovakia (Günther 2000, Wyniger 2004), Slovenia (Gogala and Gogala 1986, Gogala 2006), and United Kingdom (Anonymus 2016, Denton 2016). This is the first record for Turkey and its easternmost occurrence.

## Conclusions

As a result of this study, a total of 36 species of *Psallus* are confirmed to occur in Turkey, as detailed in the following updated checklist (see Kerzhner and Josifov 1999, Lodos et al. 2003, Önder et al. 2006, Konstantinov and Namyatova 2008, Matocq and Pluot-Sigwalt 2011, Aukema et al. 2013, Matocq et al. 2014, Dursun and Fent 2017, Çerçi and Koçak 2017). An E* indicates the species endemic for the country; non-endemic species are followed by a reference for Turkey.

## Checklist of *Psallus* from Turkey

Psallus (Apocremnus) anatolicus Wagner, 1963 E* (Wagner 1963)

Psallus (Apocremnus) anticus (Reuter, 1876) (Hoberlandt 1956)

Psallus (Apocremnus) betuleti (Fallén, 1826) (Önder et al. 2006)

Psallus (Apocremnus) skylla Linnavuori, 1994 (Matocq et al. 2014)

Psallus (Hylopsallus) perrisi (Mulsant & Rey, 1852) (Wagner 1975a, Lodos et al. 2003)

Psallus (Hylopsallus) variabilis (Fallén, 1807) (Wagner 1975a)

Psallus (Mesopsallus) ambiguus (Fallén, 1807) (Kerzhner and Josifov 1999)

Psallus (Phylidea) cerridis Wagner, 1971 E* (Wagner 1971a)

Psallus (Phylidea) collaris (Wagner, 1975) E* (Wagner 1975b)

Psallus (Phylidea) henschii Reuter, 1888 (Seidenstücker 1962)

Psallus (Phylidea) karakardes Seidenstücker, 1959 E* (Seidenstücker 1959)

Psallus (Phylidea) nigripilis Reuter, 1888 (Kerzhner and Josifov 1999, Matocq et al. 2014)

Psallus (Phylidea) quercicola (Reuter, 1904) E* (Reuter 1904)

Psallus (Phylidea) quercus (Kirschbaum, 1856) (Seidenstücker 1959)

Psallus (Phylidea) syriacus (Reuter, 1883) (Lodos et al. 2003)

Psallus (Pityopsallus) piceae Reuter, 1878 (Hoberlandt 1956)

Psallus (Pityopsallus) pinicola Reuter, 1875 (Önder 1976)

Psallus (Psallus) anaemicus Seidenstücker, 1966 (Seidenstücker 1966a)

Psallus (Psallus) apoplecticus Seidenstücker, 1966 E* (Seidenstücker 1966a)

Psallus (Psallus) asthenicus Seidenstücker, 1966 (Seidenstücker 1966a)

Psallus (Psallus) brachycerus Reuter, 1904 (Reuter 1904, Hoberlandt 1956)

Psallus (Psallus) corsicus Puton, 1875 (Konstantinov and Namyatova 2008)

Psallus (Psallus) cruentatus (Mulsant & Rey, 1852)

*Psallus* (?*Psallus*) *inancozgeni* Matocq & Pluot-Sigwalt, 2011 E* (Matocq and Pluot-Sigwalt 2011)

Psallus (Psallus) lentigo Seidenstücker, 1972 (Seidenstücker 1972)

Psallus (Psallus) lepidus Fieber, 1858 (Önder 1976)

Psallus (Psallus) milenae Josifov, 1974 (Josifov 1974)

Psallus (Psallus) mollis (Mulsant & Rey, 1852) (Kerzhner and Josifov 1999)

Psallus (Psallus) oenderi Wagner, 1976 E* (Wagner 1976)

Psallus (Psallus) oleae Wagner, 1963 E* (Wagner 1963b)

Psallus (Psallus) pardalis Seidenstücker, 1966 (Seidenstücker 1966b)

Psallus (Psallus) pseudopunctulatus Linnavuori, 1984 (Matocq et al. 2014)

Psallus (Psallus) rubinicterus Seidenstücker, 1966 E* (Seidenstücker 1966a)

Psallus (Psallus) turcicus Wagner, 1971 E* (Wagner 1971b)

Psallus (Psallus) thomashenryi sp. n. E*

Psallus (Psallus) variansvarians (Herrich-Schaeffer, 1841) (Hoberlandt 1956)

The total number of 36 species is comparable to the number of species of *Psallus* occurring in other northern Mediterranean countries such as France (31) or Italy (34), but with an important difference in the percentage of endemic species. France has no endemic species and Italy has only two endemics, both restricted to Southern Italy and/or Sicily, which make 5.9 % of the total number (Wagner 1975a, Kerzhner and Josifov 1999, Schuh 2013). In Turkey, 12 of a total of 36 species are endemic, with a percentage of 33.3 %. With the exception of Psallus (Phylidea) quercicola (Reuter, 1904), known also from the European part of Turkey, all Turkish endemic species of *Psallus* are known only from Anatolia (Seidenstücker 1959, 1962, 1966a,b, 1972; Linnavuori 1994; Lodos et al. 2003; Önder et al. 2006; Konstantinov and Namyatova 2008; Matocq and Pluot-Sigwalt 2011; Matocq et al. 2014; Dursun and Fent 2017). Moreover, one species, *Psallusdionysos* Simon & Strauss, 2014, is endemic to the Greek island of Lesbos, divided only by a narrow strait from the western coast of Anatolia (Simon and Strauss 2014) and another, Psallus (Apocremnus) cyprius Wagner, 1977, is endemic to Cyprus, not far from the southern coast of Anatolia (Linnavuori 1994). A similar high level of endemism occurs in two areas adjoining Anatolia, the Transcaucasian and the Syrian regions. In the three countries of Transcaucasia (Armenia, Azerbaijan and Georgia) 23 species of *Psallus* are known, 9 of which are endemic, with a percentage of 36 % (Zaitzeva 1968, Drapolyuk 1991, Kerzhner and Josifov 1999, Konstantinov and Namyatova 2008, Schuh 2013). In the countries of the Syrian region (Jordan, Iraq, Israel, Lebanon, Syria) 11 species of *Psallus* are known to occur, 9 of which are endemic, with a high percentage of 72.7 % (Wagner 1975a, Linnavuori 1984, Kerzhner and Josifov 1999, Carapezza 2002, Schuh 2013). In addition to the area considered above, the Palearctic region has two more centers of endemism for the genus *Psallus*: a minor one in the western Mediterranean (four endemic species in Spain, two in Italy, and one in Algeria) (Wagner 1975a, Kerzhner and Josifov 1999, Konstantinov and Namyatova 2008, Schuh 2013, Pagola-Carte 2017) and a major one in the Palearctic Far East of Asia (seven endemic species in the Far East of Russia, seven in the Korean Peninsula, seven in China, and nine in Japan) (Josifov 1983, Zheng and Li 1990, Li and Zheng 1991, Vinokurov 1998, Kerzhner and Josifov 1999, Yasunaga and Vinokurov 2000, Duwal et al. 2012, Schuh 2013, Duwal and Lee 2015). These data show the high relevance of the Syro-anatolian-transcaucasian region as center of origin of the speciation process of the genus *Psallus* for the Western Palearctic.

## Supplementary Material

XML Treatment for Psallus thomashenryi

XML Treatment for Atomoscelis onusta

XML Treatment for Plagiognathus (Plagiognathus) marivanensis

XML Treatment for Psallus (Psallus) lucanicus
